# Machine learning accelerated high-throughput screening of zeolites for the selective adsorption of xylene isomers†

**DOI:** 10.1039/d2sc03351h

**Published:** 2022-10-24

**Authors:** Daniel Hewitt, Tom Pope, Misbah Sarwar, Alessandro Turrina, Ben Slater

**Affiliations:** Department of Chemistry, University College London 20 Gordon Street London WC1E 6BT UK dan.hewitt.15@ucl.ac.uk b.slater@ucl.ac.uk; Johnson Matthey Technology Centre Sonning Common, Reading RG4 9NH UK; Johnson Matthey Technology Centre Chilton, P.O. Box 1, Belasis Avenue Billingham TS23 1LB UK

## Abstract

The production of widely used polymers such as polyester currently relies upon the chemical separation of and transformation of xylene isomers. The least valuable but most prevalent isomer is *meta*-xylene which can be selectively transformed into the more useful and expensive *para*-xylene isomer using a zeolite catalyst but at a high energy cost. In this work, high-throughput screening of existing and hypothetical zeolite databases containing more than two million structures was performed, using a combination of classical simulation and deep neural network methods to identify promising materials for selective adsorption of *meta*-xylene. Novel anomaly detection techniques were applied to the heavily biased classification task of identifying structures with a selectivity greater than that of the best performing existing zeolite, ZSM-5 (MFI topology). Eight hypothetical zeolite topologies are found to be several orders of magnitude more selective towards *meta*-xylene than ZSM-5 which may provide an impetus for synthetic efforts to realise these promising materials. Moreover, the leading hypothetical frameworks identified from the screening procedure require a markedly lower operating temperature to achieve the diffusion seen in existing materials, suggesting significant energetic savings if the frameworks can be realised.

## Abstract 2

A combination of machine learning and high throughput simulation has identified several potential zeolite structures that appear to outperform the leading commercially used material and explained the key factors for high selectivity.

## Graphical abstract

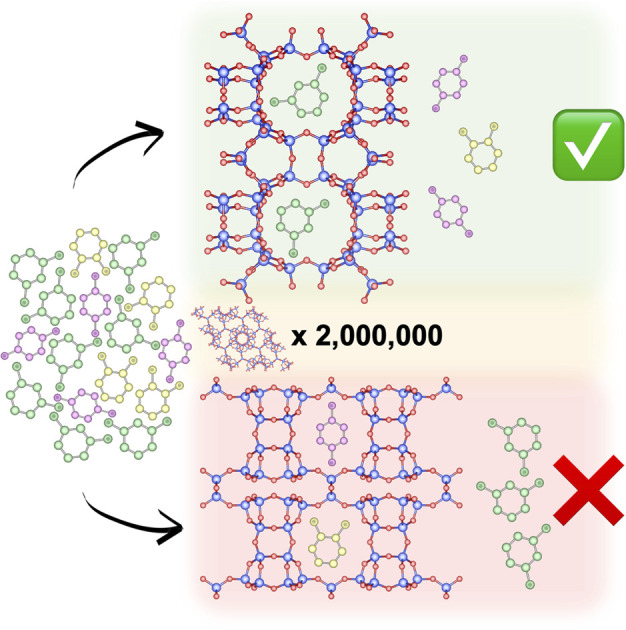

## 1 Introduction

The separation of benzene and its derivatives using current distillation methods accounts for 50 GW of global energy usage annually,^1^ where one particularly energy intensive process is the isomerisation and separation of xylene isomers. The identical molecular weights, similar boiling points, and closely resemblant structures make this extremely challenging. However, the application of sorbent and catalytically active materials can increase the yield of the most commercially important products, reducing both the demand for raw materials and the associated energy cost of production *via* distillation columns. Are the currently deployed catalysts already optimally efficient for this key process or is there scope to improve the yield further by identifying untried existing materials or yet-to-be synthesized materials? Here, we address this question using novel computational approaches and seek to identify the key properties required for high selectivity of particular isomers.

We seek to identify the optimal zeolite topology for the isomerisation of *meta*-xylene from a mixture of its isomers by tapping into experimental and hypothetical databases, where the latter could reveal yet-to-be-synthesised materials with superior performance to existing materials. Hitherto undiscovered textural properties responsible for enhanced performance could then be used as design criteria for the next generation of industrial catalyst.

**Fig. 1:**
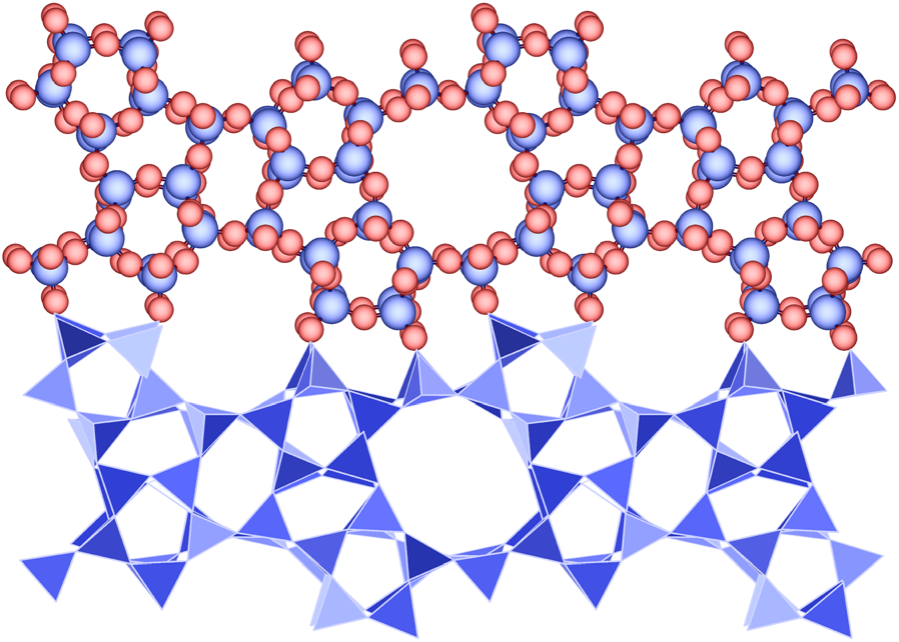
Structure of the siliceous form of the zeolite MFI shown as (top) ball and stick form, atoms shown with their van der Waals radii and (bottom) polyhedral form to show the silica tetrahedra. The structure is viewed along the [010] axis and shows a fragment cut from the extended solid. Silicon atoms and tetrahedra are shown in blue, and oxygen atoms are shown in red.

Zeolites are a class of nanoporous materials which have been widely investigated previously, as sorbents and as catalysts for isomerisation reactions.^2–5^ Zeolites are commonly used for separation and catalysis processes due to their high thermal stability, shape selectivity and availability of catalytically active sites. A variety of shape selectivity is possible due to the range of topologies in the zeolite family, coupled with their highly tunable pore sizes that facilitates discrimination of mixtures of molecules or isomers.^6^ There are a number of different zeolite compositions, the most commonly used materials are those based on aluminosilicates. However, the aluminosilicates typically feature disordered aluminium and extra-framework or intra-framework charge compensating species that adds structural complexity to the modelling process. To simplify the modelling of large numbers of frameworks, a sub-class of zeolites are used, framework silicates of formula SiO_2_, that consist of edge-sharing tetrahedra. Oxygen atoms occupy the vertices of the tetrahedra and silicon atoms reside in the centre. An example is presented in Fig. 1 which shows the MFI structure and its characteristic 10 membered rings which provide access to the three-dimensional channel system.

Commercial applications of zeolites exploit their shape-selectivity arising from the characteristic pore network. The fluid catalytic cracking process typically uses a zeolite catalyst with the FAU topology with a high silicon concentration, while the methanol to olefin (MTO) process uses a zeolite in its silica-aluminophosphate (SAPO) form, SAPO-34,^7^ with the CHA topology. The former process benefits from the relatively large pores and high internal surface area of FAU, facilitating diffusion of larger organic products.^8^ In contrast, CHA sees use in the MTO process due to its higher selectivity toward short-chain olefins promoted by its small pore openings, which connect cages large enough to accommodate intermediates.^9^ Zeolites have also been proposed as promising materials for the *meta*-xylene isomerisation process.^2^ Topologies with pore geometries ideally suited to selectively hindering the transport of *m*-xylene increases its chance of being near a catalytically active proton site, and thus promoting its conversion to *para*-xylene.

**Fig. 2:**
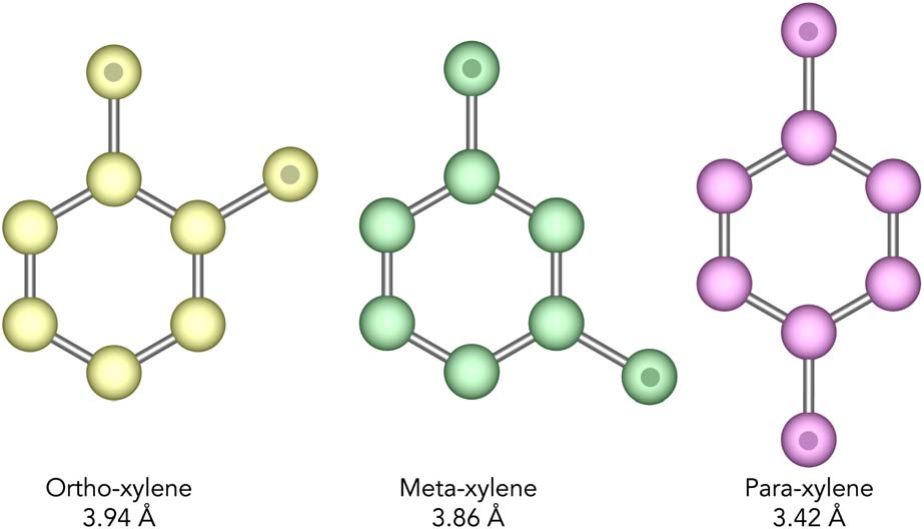
United-atom representation of the structure of the xylene isomers, with CH_3_ groups indicated by a spot. The shortest dimension in the plane of the molecule for each isomer is given beneath their name and the variance between the isomers of just 0.5 Å emphasises how discriminating the catalyst needs to be. *Ortho*-, *meta*-, and *para*-xylene will hereafter be referred to as *o*-, *m*-, and *p*-xylene respectively.

Xylenes are isomers of dimethyl benzene which are produced predominantly by the catalytic reforming of crude oil. Typically, after distillation, a mixture of xylenes will contain *ortho*-, *meta*-, and *para*-xylenes (see Fig. 2) in a ratio of 0.24, 0.53, and 0.23 respectively.^10^ Of these, *o*- and *p*-xylene have commercial value as the raw material in the manufacture of phthalic anhydride, and poly(ethylene terephthalate) respectively, with *p*-xylene being the most valuable of the two.^4,11^*M*-xylene is considered the least commercially valuable of the three isomers and so the separation of these isomers is an important chemical process. Specifically, the isomerisation of *m*-xylene to produce *p*-xylene is of particular importance.

Screening for promising materials experimentally involves a high monetary and temporal cost, due to the difficulty associated with preparation and characterisation of samples. That bottle-neck is further exacerbated for hypothetical frameworks where the preparative mechanism may not be clear. Indeed, the synthetic routes for hypothetical zeolites are so elusive as to be called the zeolite conundrum.^12^ High-throughput computational screening offers a comparatively inexpensive alternative to practical experiment subject to the veracity of predicted behaviours.^13,14^ Zeolites have been screened for their application in numerous processes by computer simulation, such as the hydroisomerisation of alkanes with 18–30 carbon atoms.^15^ Combining modelling techniques with machine learning methods allows for a reduction in simulation time on the scale of hundreds of days on a single CPU into seconds. Here, we use this strategy to rapidly screen experimental^16^ and hypothetical^17,18^ databases of zeolites in a hierarchical manner, for their potential application in the *m*-xylene isomerisation reaction.

A subset of 4764 metal–organic frameworks (MOF) from the CoRE-MOF database^19^ have previously been screened in a study by Qiao *et al.*,^5^ as molecular sieves in order to filter out *para*-xylene. Ortiz *et al.*^3^ recently carried out detailed assessment of nine industrially relevant zeolite topologies, to establish the competition between enthalpic and entropic effects in the transport of the xylene isomers. Here, we build upon this work and screen over two million structures (known and hypothetical frameworks) to identify whether there are other topologies of zeolite which could outperform industrially used catalysts for the isomerisation of *meta*-xylene.

## 2 Methods

In this work, two databases of zeolite structures were screened for their application in the *m*-xylene isomerisation reaction. The international zeolite association (IZA) database contains, as of March 2022, 255 distinct topologies with known synthetic routes.^16^ The Deem database is a set of hypothetical zeolites which contains over two million structures, approximately 330 000 of which are below +30 kJ mol^−1^ of alpha-quartz as assessed by the Sanders–Leslie–Catlow (SLC) force field, a cutoff that seeks to capture the structures which are most synthesizable.^17,18,20^ The high-throughput screening of these two databases allows for the identification of highly selective materials which could either be synthesised, or provide insight into key structure–property relationships which can inform future synthetic design.

### 2.1 Textural property screening

The textural characteristics of pore limiting diameter (PLD) and largest cavity diameter (LCD) were calculated for the IZA^16^ and Deem^17,18^ databases of structures using the Zeo++ software.^21^ These were used as an initial screen for both databases in order to remove structures which would not accommodate the xylene isomers. A PLD cut-off of 4.0 Å was chosen for this purpose, as it was the smallest value that could be used while accommodating the bulkiest xylene isomer (*ortho*-xylene, with a shortest dimension of 3.94 Å as shown in Fig. 2). Applying this screen to over 330 000 structures from both the IZA and Deem databases yielded 66 177 structures, 98 of which were from the IZA database and 66 079 from the Deem database. Initially calculations were run for just the 98 structures from the IZA database. The topology identified to have the highest selectivity from these existing zeolites was used as a benchmark by which to compare the hypothetical structures to.

### 2.2 Screening IZA database structures for uptake

The 98 filtered structures from the IZA database were assessed for their competitive adsorption properties by use of continuous fractional component Monte Carlo (CFC-MC)^22^ simulations in the grand canonical ensemble as implemented in RASPA,^23^ at 523 K and 15 bar which typify the experimental conditions for this process.^24–26^ The composition of the xylene mixture at this temperature and pressure was adapted from the work of Caro-Ortiz *et al.* where they studied the same process in nine experimentally realised framework topologies.^3^ These simulations were run for 20 000 initialisation cycles, 50 000 equilibration cycles, and 50 000 production cycles. The relatively brief simulations have been benchmarked against far longer simulations with 100 000 initialisation cycles, 500 000 equilibration cycles, and 500 000 production cycles, where the same loadings per component were obtained with a 90% reduction in computational cost. Example input files are provided in the ESI.†

#### 2.2.1 Simulation details

The TraPPE-zeo all-silica force field^27^ and the TraPPE-UA force field^28,29^ were used throughout this work in order to model the interactions between the framework and guests, as well as guest–guest and framework–framework interactions. All atoms were modelled using Lennard–Jones interactions, truncated with a cut-off distance of 14.0 Å, with long-range Lennard–Jones contributions estimated *via* tail corrections. As the TraPPE-UA force field models CH_*x*_ as single uncharged interaction sites, electrostatic interactions are not considered in this work; similarly, framework flexibility is not explicitly taken into account. These assumptions were considered reasonable as this work predominately focuses on adsorption calculations, while framework flexibility is suggested to have a larger influence on transport properties;^27^ comparison of transport properties to experiment is often challenging due to the large scatter in data, especially across different studies^27^ and so these simulations were considered a reasonable first approximation.

The top candidates from the adsorption calculations were judged on their loading and selectivity. Selectivity was defined in this work by the following equation, based on the work of Bae *et al.*:^30^1
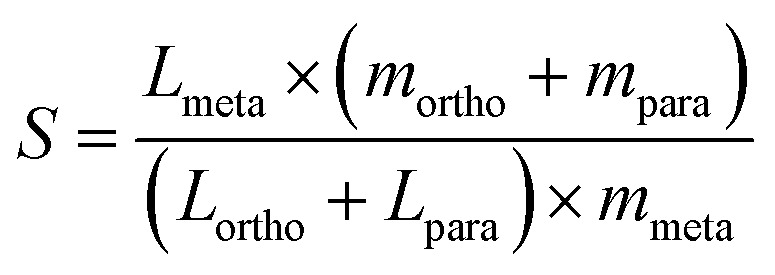
where *L*_*x*_ refers to the loading of isomer *x*, and *m*_*x*_ refers to the mol fraction of isomer *x*. The structure from the IZA database with the highest selectivity was found to be MFI, with a selectivity value of 12.19 (approximately 1 × 10^1.086^) and this was used as the benchmark to assess hypothetical structures from the Deem database. Approximating selectivity by use of loading values allowed for a significant saving in computational time over brute-force molecular dynamics simulations. The assumption this strategy makes is that a higher loading value for an isomer is strongly correlated with a greater retention of the given isomer within the framework. In turn, this slower transport leads to a greater probability of an isomer being near a catalytic site and undergoing isomerisation.^15^

Here zeolite structures are screened only in their siliceous form. Although we note that materials used industrially are aluminosilicates, in the absence of a database of realistic forms of such materials, we seek only to identify topologies of structures which may favour a greater selectivity for this process.

### 2.3 Screening Deem database structures

As the IZA database contained only 98 structures with a sufficiently large PLD to accommodate the xylene isomers, a purely classical simulation approach was practicable in a short amount of time. The Deem database on the other hand contained 66 079 structures with a PLD greater than 4.0 Å. In order to determine the viability of these materials for the *m*-xylene isomerisation reaction, a combination of classical simulation and machine learning methods were applied in order to reduce the total compute time required.

#### 2.3.1 Classical simulation of xylene uptake

CFC-MC simulations as outlined in Section 2.2.1 were run for 8 hours for all 66 079 structures from the Deem database in order to generate the initial dataset (in this time only 2695 simulations had completed the predetermined number of steps for convergence). Using the data set comprised from only the simulations which had finished by this time (2695 data points), a neural network (NN) was trained for binary classification of selectivity, with one class representing structures whose selectivity was greater than that of the leading IZA candidate, the MFI structure.

Simulations were incrementally run for a further hour from the point at which they had been stopped. After each hour data was extracted and a new NN model was trained using the full dataset obtained after this time period (after 9 hours this contained 5000 data points). This was repeated incrementally until no significant further improvements were made on the model after data generation for longer time periods. It was determined that after a total of 10 hours, at which point 7802 data points had been collected, the NN was suitable for deployment, showing an accuracy and recall value of over 80%. Continued data collection and model training for up to a total of 48 hours of simulation time provided no substantial improvement in the predictive ability of the model.

#### 2.3.2 Machine learning predictions

In order to train a neural network, one must provide a target quantity, and numerical descriptors that can adequately represent the desired quantity. Both neural networks in this work were trained using the same descriptor set which is laid out below, and the data was split into a training, validation, and blind test set in a stratified 70%, 20%, 10% split respectively.

Many studies in the high-throughput screening field of nanoporous materials use basic textural characteristics such as PLD, LCD, density and accessible surface area (ASA) as descriptors.^5,6,31^ This combination of descriptors has been shown to be incredibly powerful, especially for packing problems.^5^ However, a recent study by Krishnapriyan *et al.* showed how the combination of these textural features along with topological descriptors called persistence images, obtained from persistent homology calculations, can significantly increase the predictive accuracy of such models (see ESI†).^31^

In this work a combination of these two sets of descriptors was employed. The textural characteristics PLD and LCD were calculated using Zeo++,^21^ while the remaining textural characteristics such as accessible surface area were calculated using PoreBlazer.^32^ Persistence images were calculated using the Python packages ‘Diode’,^33^ ‘Dionysus',^34^ and ‘PersIm’.^35^ After testing different parameters, a resolution for the persistence images of 50 × 50 pixels was chosen, with the *x* and *y* axes scaled by the maximum birth and persistence values across all structures respectively. A spread of *σ* = 0.2 for these images was also used. More details on the descriptor set used can be found in the ESI.†

In order to determine a model's performance for binary classification tasks, two key metrics were used: accuracy and recall. Accuracy is defined as the percentage of examples which are correctly classified:2
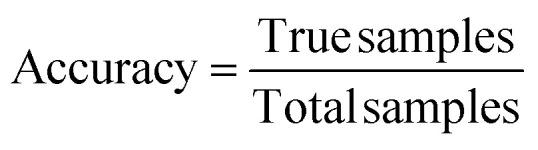


Recall is defined as the percentage of true positives which were correctly identified:3
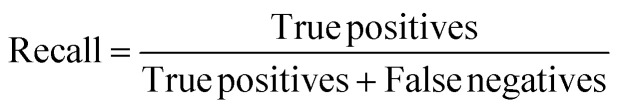


Maximising recall corresponds to identifying as many leading frameworks as possible, while maximising accuracy allows us to reduce our search space by eliminating structures with low selectivity. Attempting to maximise one can lead to a reduction in the other, and so here we sought to train a model with a compromise between the two metrics, with more weight given to a higher recall value so as not to miss out on any promising structures.

#### 2.3.3 Selectivity classification model

As detailed in Section 2.3.1, simulation data after 10 hours yielded a dataset containing loading and selectivity data for 7802 structures. As one class in the binary classification task was in an extreme minority (5% of the dataset), anomaly detection methods were used in order to provide more weight to the underrepresented class, preventing the model from overfitting to the majority class.^36^ The NN trained using this dataset performed remarkably well, showing an accuracy of 83% and a perfect recall of 100%. When tested on an unseen part of the dataset, the model showed an accuracy of 83% and a recall of 89%.

In order to further validate the predictive power of this model, a much larger dataset was generated. Simulations were continued until they had all run for 48 hours, at which point the total dataset contained the results from 42 183 completed simulations. Applying the NN model to the blind dataset of simulations that finished between 10 and 48 hours showed an accuracy of 84% and a recall of 98%. Running these classical simulations took 97.4 years of CPU time on a single core (hereby referred to simply as CPU time). Accounting for the 10 hours per calculation that had already been run for these structures, using the machine learning predictions to filter this set of structures, followed by classical simulations on the 10% of structures identified to be highly selective, could have resulted in a time saving of 47 years of CPU time for these calculations, effectively halving the overall time and energy cost for computation.

#### 2.3.4 Loading classification model

In order to reduce the search space for optimal structures further, classification models to predict loading were trained concurrently with those predicting selectivity. Here the binary classification model categorised structures into low and high loading bins, where the target quantity was chosen to be the sum of the loading values for all three xylene isomers. The low bin contained all structures with a total loading below 0.2 mol kg^−1^. By choosing total loading of all xylene isomers as the target value, the model did not have to discriminate against structures which preferentially adsorb one isomer over another. Choosing this target rather than the loading of just the *m*-xylene isomer provided an increase in model accuracy from 90% to 93% and recall from 93% to 96%. These findings can be rationalised as the textural and topological descriptors are more informative in determining a structure's uptake than their selectivity, as loading is more dependent on descriptors such as the accessible volume. This can be seen quantitatively in the higher accuracy and recall of the loading model compared to the model predicting selectivity.

Similar to the classification model for selectivity, this NN was tested on an unseen part of the dataset, showing an accuracy of 90% and a recall of 95%. When applied to the blind dataset from 33 849 simulations that finished between 10 and 48 hours of compute time, the model showed an accuracy of 94% and a recall of 95%. Of these 34 381 structures, 31 649 possessed a total xylene loading greater than 0.2 mol kg^−1^ meaning the NN was able to correctly predict 30 805 of these. None of the 844 structures with high loading, but misclassified as low loading, possessed a high selectivity.

### 2.4 Data validation

In order to verify the results of the neural network model, CFC-MC simulations were run on the structures which were predicted to be highly selective using the same methods as outlined in Section 2.2.1. Of the top candidates identified, only those frameworks with channel networks percolating in greater than one dimension were considered further. The Zeo++ program was used to assess dimensionality, with a probe of radius 2.0 Å.^21^ Only structures with 2 or 3D channel systems were assessed further, as frameworks with 1D channels are of little interest commercially due to their associated reduced activity and flow rates. Longer CFC-MC simulations were then carried out on these structures using 100 000 initialisation cycles, 500 000 equilibration cycles, and 500 000 production cycles in order to obtain better statistics for the adsorption properties. A comparison of the statistics between these calculations and the initial results showed good agreement between the two, with an average difference of 3% in the total loading values across all top performing structures, and in complete agreement with the initial ranking of structures.

Molecular dynamics (MD) simulations to assess the diffusivity of xylene mixtures were carried out using the RASPA software package on the leading candidates that showed high dimensionality, high loading, and a selectivity greater than that of MFI (MD simulations on MFI were used as a benchmark for comparison). These simulations used a temperature of 1223 K, higher than that in the CFC-MC calculations, in order to promote diffusion of the molecules across high energy barriers. A comparison of the mean-square displacement (MSD) for each adsorbate was used as a metric to validate the selectivity predicted by CFC-MC.

## 3 Results and discussion

### 3.1 Classical simulations

Classical simulation data for 42 183 hypothetical and 98 experimentally realised zeolites was generated as outlined in Section 2, along with corresponding textural characteristics as outlined in Section 2.3.2.

**Fig. 3:**
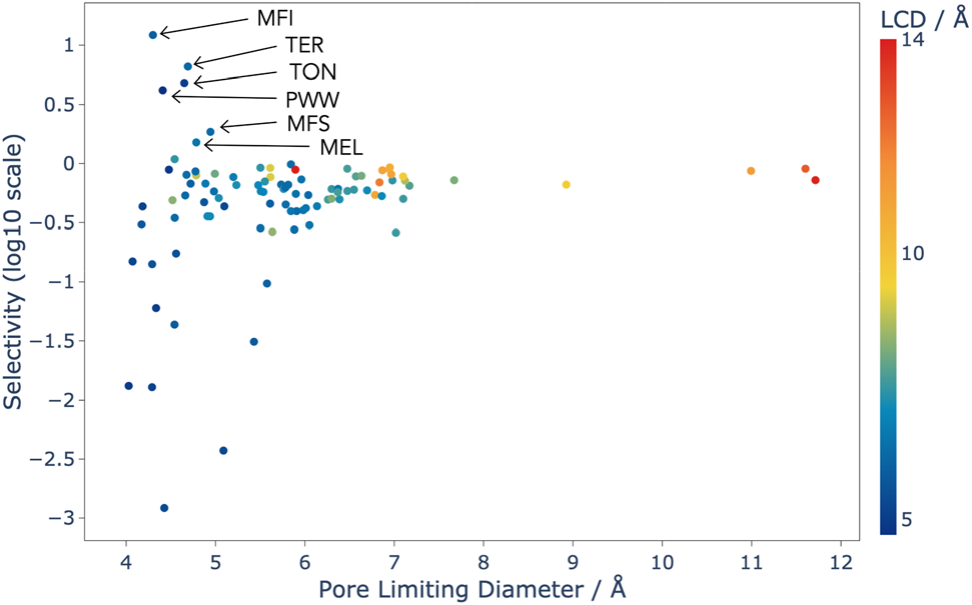
Selectivity as a function of PLD for the 98 experimentally realised zeolite topologies that were screened using CFC-MC, and coloured by LCD. Selectivity is defined in Section 2.2.1. Some highly selective structures have been labelled with their three letter topology code given by the IZA.

**Fig. 4:**
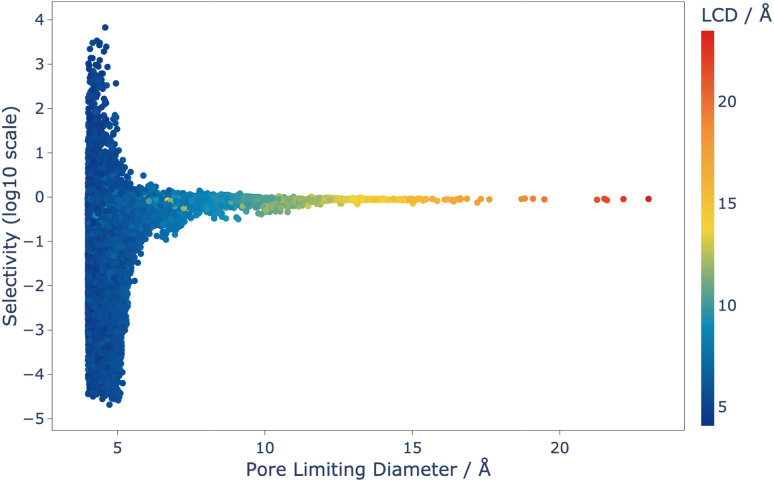
Selectivity as a function of PLD for the 42 183 structures that were screened using CFC-MC, and coloured by LCD. Selectivity is defined in Section 2.2.1.

Analysis of this data shows that the limit of selectivity for structures from the IZA database is approximately 12.19 (or 1 × 10^1.086^) (see Fig. 3), whereas hypothetical structures from the Deem database are able to surpass this by over two orders of magnitude (see Fig. 4) emphasising the latent performance which could be realised. It is also noteworthy that structures with the highest selectivity have PLD values between 4.0 and 5.5 Å, which is expected, as this shows that the most potent mechanism for selectivity is predominately related to steric discrimination of the single isomers in channels.

The results for the IZA structures are in good agreement with experimental studies, with MFI and TON both having high selectivity.^37^ MFI is a topology of zeolite that is already used industrially for the xylene isomerisation reaction, and these results predict that it is the best choice from the list of currently known zeolite topologies. Our models thus show that there is currently no existing zeolite which is superior to MFI for this process, and so any advance in the efficiency of this process is likely to come from an as-yet unrealised topology.

Using MFI as a benchmark, a set of hypothetical structures with sufficiently high selectivity to be of interest commercially were identified. From the set of 42 183 structures that were able to be computed within 48 hours using these classical simulation techniques, only 58 showed high enough selectivity and loading to be of further interest, which is just 0.14% of the set.

**Fig. 5:**
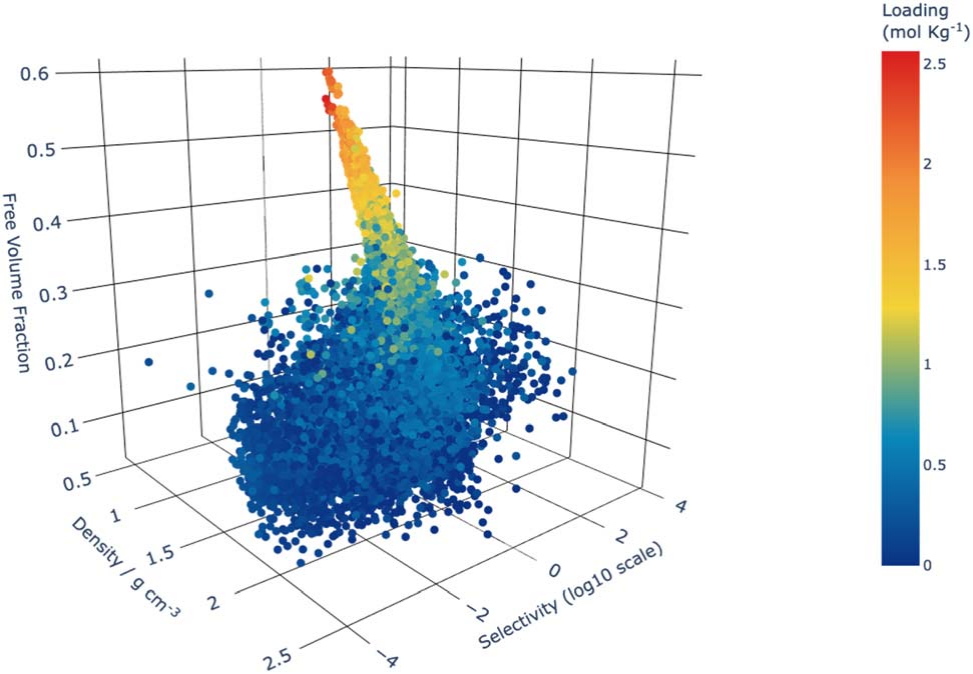
Selectivity as a function of density and free volume fraction for the 42 183 structures that were screened using CFC-MC, and coloured by the total loading. Selectivity is defined in Section 2.2.1.

Fig. 5 shows that the structures with the highest total loading of all xylene isomers were those with the greatest free volume fraction (FVF), as expected. Structures with these attributes are also shown to more frequently feature low densities and large pore limiting diameters, which makes them unlikely as practical candidates for this separation task because of their poor steric discrimination ability. Confirmation of this inference is evidenced by comparing the total loading of the xylene isomers to selectivity as shown in Fig. 5.

By extension of these trends, we are able to identify the “Goldilocks” area of structural features where materials may have both high selectivity and high loading. Identification of these structure–property relationships allows for targeted future investigation of optimal catalysts for this process as those structures with: (i) PLD between 4.0 and 5.5 Å, (ii) FVF greater than 0.1, (iii) density lower than 2 g cm^−3^.

### 3.2 Neural network predictions

The classification NN model outlined in Section 2.3.3, trained to predict structures with selectivities greater than MFI, was applied to the remaining 23 896 hypothetical structures for which direct classical simulations took over 48 hours to complete. Applying the NN model to the unlabeled dataset reduced the number of potentially highly selective structures from 23 896 to 5376 providing an 76% reduction in the search space for highly selective materials. The model was trained to have high recall at a detriment to accuracy in order to identify almost all of the optimal structures while drastically reducing the number of materials that needed to be examined with further calculations.

**Fig. 6:**
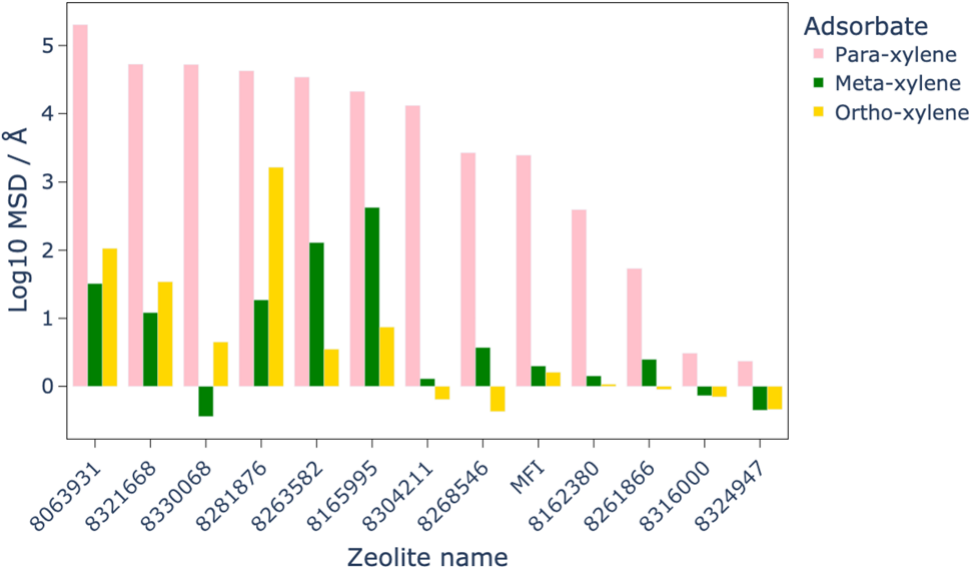
Comparison of total order N MSD in the *x*, *y*, and *z* directions for each xylene isomer in the 12 most optimal structures, as well as for MFI as a benchmark. The total order N MSD has been again log 10 transformed for clarity.

In order to reduce the search space for optimal materials further, the NN model trained to classify structures based on their total loading values was applied to the remaining set of 5376 structures. The model classified 4838 of the 5376 structures in the high loading bin, which show that they are of further interest.

CFC-MC calculations as outlined in Section 2.2 were then performed on the set of structures predicted to possess both high selectivity and loading by the neural networks; the results of this showed that 23 of the 4838 structures had selectivity greater than MFI, which is 0.10% of the total number of structures before filtration by the neural network (this value is in good agreement with the percentage of structures that were determined to be highly selective through purely classical simulations).

Assuming that these simulations would have taken at a minimum 48 hours to successfully complete the number of cycles outlined in 2.2, the total CPU time for these classical simulations would have been 130 years. Accounting for the 10 hours these simulations were run for in order to generate the dataset for the NN model as well as the time taken for the classical simulations to complete for the structures predicted to be highly selective, this method was able to save 92 years worth of CPU time in total.

### 3.3 Refining optimal structures

By combining the optimal structures predicted using purely classical simulation methods (58 structures) with the structures identified through a combination of machine learning and classical simulation (23 structures), it was possible to identify 81 highly selective structures for further examination. An additional refinement of promising structures was achieved by focusing only on those with multi-dimensional channel systems, which led to a reduction of the 81 structures to 12 of especial interest. Of this final set, 3 of the 12 structures contained three dimensional channel systems.

### 3.4 Analysis of transport properties

MD simulations were used to assess the diffusion of the xylene isomers throughout the structures of the 12 most promising candidate structures, as well as MFI as a benchmark. The results of these simulations, shown in Fig. 6, identified eight of the twelve structures which allowed greater diffusion of the isomers than MFI, with all structures showing higher diffusion of *para*-xylene than *ortho*- or *meta*-xylene, which makes them viable candidates for this separation process.

MD simulations for the eight materials which showed greater diffusion than MFI at 1223 K were run at lower temperatures in steps of 50 K. By comparison of the MSD of these structures at lower temperatures to that of MFI at 1223 K, we are able to quantitatively show a possible energy saving by finding the lowest temperature at which these structures outperform the diffusion of xylene isomers in MFI. The results of these calculations showed that the leading candidate, PCOD-8063931, showed comparable diffusion of the xylene isomers at 723 K than through MFI at 1223 K.

### 3.5 Analysis of promising materials

In order to further examine the adsorption behaviour of the top performing structures, adsorption profiles were generated for all isomers within each structure using in-house Python code.^38^ This tool allowed extraction of binding positions from the classical Monte Carlo simulations in order to generate a probability grid for each isomer's position; positions were mapped onto 0.1 Å grid points, and only those over a threshold probability were displayed.

**Fig. 7:**
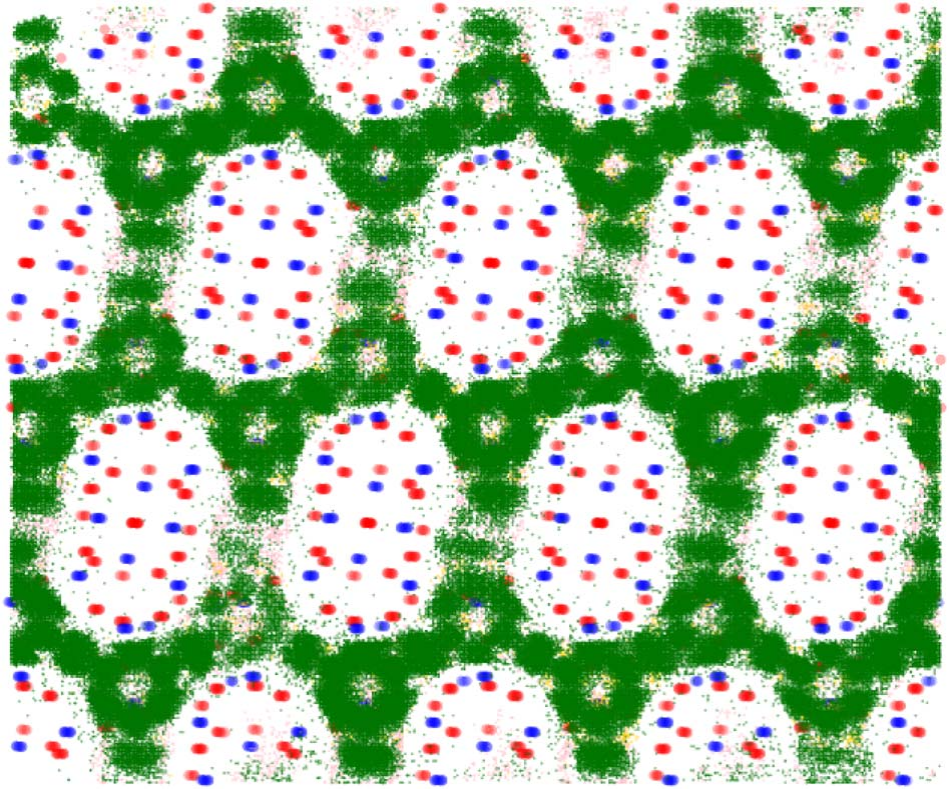
Visualisation of the statistically most preferable adsorption sites for each xylene isomer within the framework PCOD-8063931. The framework atoms silicon and oxygen are shown in blue and red respectively, while the adsorbates *ortho*-, *meta*-, and *para*-xylene are shown in gold, green, and pink respectively.

**Fig. 8:**
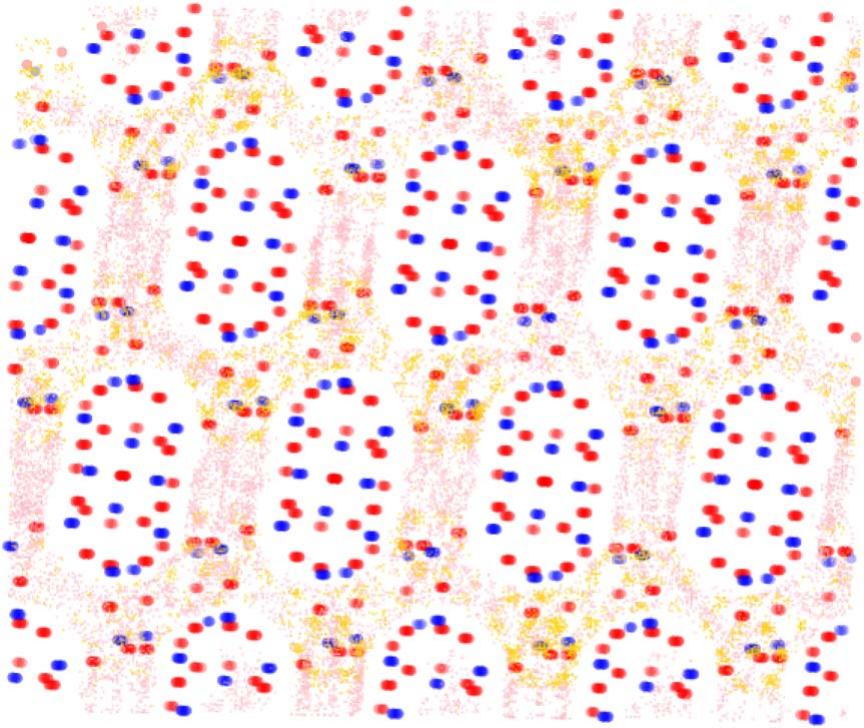
Visualisation of the statistically most preferable adsorption sites for *ortho*- and *para*-xylene in the framework PCOD-8063931. The framework atoms silicon and oxygen are shown in blue and red respectively, while the adsorbates *ortho*- and *para*-xylene are shown in gold and pink respectively.

By inspection of Fig. 7 and 8 it can be seen that in PCOD-8063931, *m*-xylene gets trapped in a strong binding site at the intersection of the channels. This site is able to perfectly accommodate the *m*-xylene adsorbate due to the 120° angle of the intersection. Although this site is also the most preferable for *ortho*-xylene to adsorb, *meta*-xylene is adsorbed far more favourably which is reflected in its 8.5 kJ mol^−1^ lower heat of adsorption.

It was also determined that *para*-xylene sits preferentially in the channels of the structures rather than the intersections, as is shown in Fig. 8. Although *para*-xylene is accommodated in the channels better than the intersections, its adsorption is still disfavoured overall, which is consistent with its 14 kJ mol^−1^ greater heat of adsorption compared to *meta*-xylene. This energetic preference is confirmed by the results of the molecular dynamics simulations, with a much higher MSD value for *para*-xylene than for *ortho*- or *meta*-xylene showing its ability to readily diffuse through the framework. This increased diffusion ability is due to the more cylindrical shape of *para*-xylene allowing it to move through the channels of the structure, while not suffering too great an energy penalty for traversing channel intersections. On the other hand, *m*-xylene fits snugly at channel intersections but it has a high energy penalty for diffusing through channels.

## 4 Conclusions

A powerful combination of classical simulation and cutting-edge machine learning techniques facilitated the high-throughput screening of over two million zeolite structures, with an estimated time saving of 118 years of CPU time over traditional screening procedures. Our novel methodology in determining these results can be applied to future high-throughput screening work with ease, facilitating rapid data generation with a high accuracy and retention rate of optimal structures.

Remarkably, exploration of this vast search-space resulted in the identification of just eight materials whose topologies are predicted to be superior to the current industry standard for the *meta*-xylene isomerisation process, the ZSM-5 zeolite with the MFI topology. These results suggest that as the best existing material is already in use, a significant improvement in the efficiency of this process can only be achieved through the realisation of the hypothetical frameworks: PCOD −8 063 931, −8 321 668, −8 330 068, −8 281 876, −8 263 582, −8 165 995, −8 304 211 and −8 268 546 (CIF files are given in the ESI†). Furthermore, we predict that the vastly superior performance of PCOD-8063931 could result in a potential 40% reduction in reaction temperature for this process, promising higher yields at a lower energy cost. Further improvements in performance may be possible in aluminosilicate forms of these structures and research is underway in this direction.^39^ We hope that the potential gains of the top performing structures identified from the screening work motivates experimental research groups to take up the challenge of synthesising zeolites with pore networks similar to those leading structures that we have identified.

## Data availability

Simulation results and top structures are available through the GitHub link in the ESI.†

## Author contributions

Daniel Hewitt: conceptualisation, data curation, formal analysis, investigation, methodology, software, validation, visualization, writing – original draft, writing – review and editing Tom Pope: methodology, software, writing – review and editing Misbah Sarwar: funding acquisition, project administration, writing – review and editing Alessandro Turrina: funding acquisition, project administration, writing – review and editing Ben Slater: conceptualisation, funding acquisition, project administration, supervision, writing – review and editing.

## Conflicts of interest

There are no conflicts to declare.

## Supplementary Material

SC-013-D2SC03351H-s001
