# Supplementary material for: Machine learning accelerated high-throughput screening of zeolites for the selective adsorption of xylene isomers
Source: Chem Sci. 2022 Oct 24;13(44):13178–86. doi: 10.1039/d2sc03351h (PMC9667951; doi:10.1039/d2sc03351h)
Supplement: SC-013-D2SC03351H-s001 [file SC-013-D2SC03351H-s001.pdf]

Electronic Supplementary Material (ESI) for Chemical Science.
This journal is © The Royal Society of Chemistry 2022

Supporting information for "Machine learning accelerated high-throughput screening of zeolites for the selective adsorption of xylene isomers"

Daniel Hewitt,[a*] Tom Pope,[a] Misbah Sarwar,[b] Alessandro Turrina[c] and Ben Slater[a*]

October 10, 2022

Contents

1 Top performing hypothetical structures **2**

1.1 Comparison of MFI and MEL 5

1.2 Loading and selectivity data 7

2 Computational Details **7**

2.1 GCMC 7

2.2 Molecular Dynamics 7

3 Neural Network details **10**

3.1 Model architecture 10

3.2 Classification model 12

3.3 Regression model 13

3.4 Descriptors 13

Corresponding authors: dan.hewitt.15@ucl.ac.uk, b.slater@ucl.ac.uk

[0a] *Department of Chemistry, 20 Gordon Street,University College London, London, WC1E 6BT, UK*

[b] *Johnson Matthey Technology Centre, Sonning Common, Reading, RG4 9NH, UK*

[c] *Johnson Matthey Technology Centre, Chilton, P.O. Box 1, Belasis Avenue, Billingham, TS23 1LB, UK*

1 Top performing hypothetical structures

All CIF and adsorption profile html files for the top eight performing hypothetical structures are available through this link. Loading and selectivity data is available in Tables 1 - 3.

We have included here the structures of the three top performing real and hypothetical structures, MFI, TON, and TER, as well as PCOD-8063931, -8321668, and -8330068.

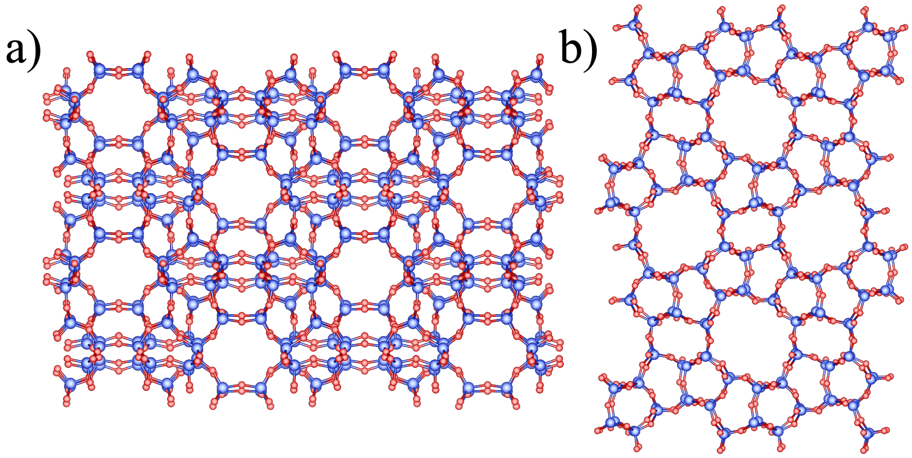

Figure 1: Structure of a supercell of the topology MFI viewed along the [1 0 0] and [0 1 0] axes respectively, rotated for ease of inspection. Silicon is shown in blue, and oxygen in red.

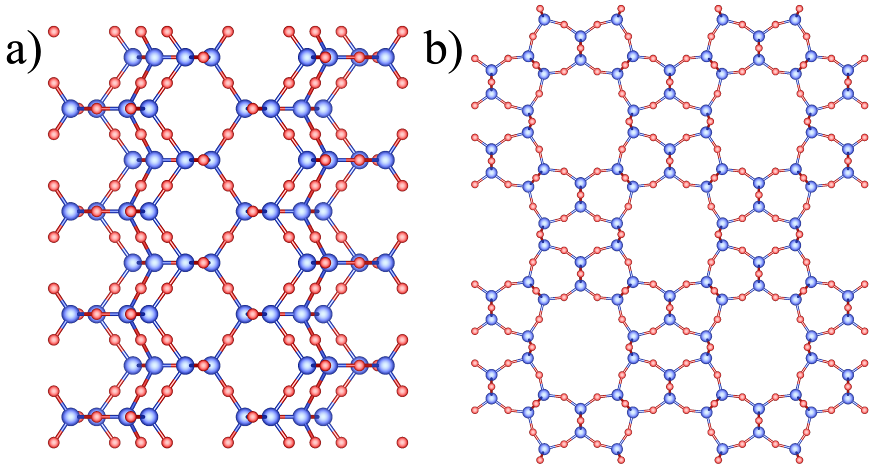

Figure 2: Structure of a supercell of the topology TON viewed along the [1 0 0] and [0 0 1] axes respectively, rotated for ease of inspection. Silicon is shown in blue, and oxygen in red.

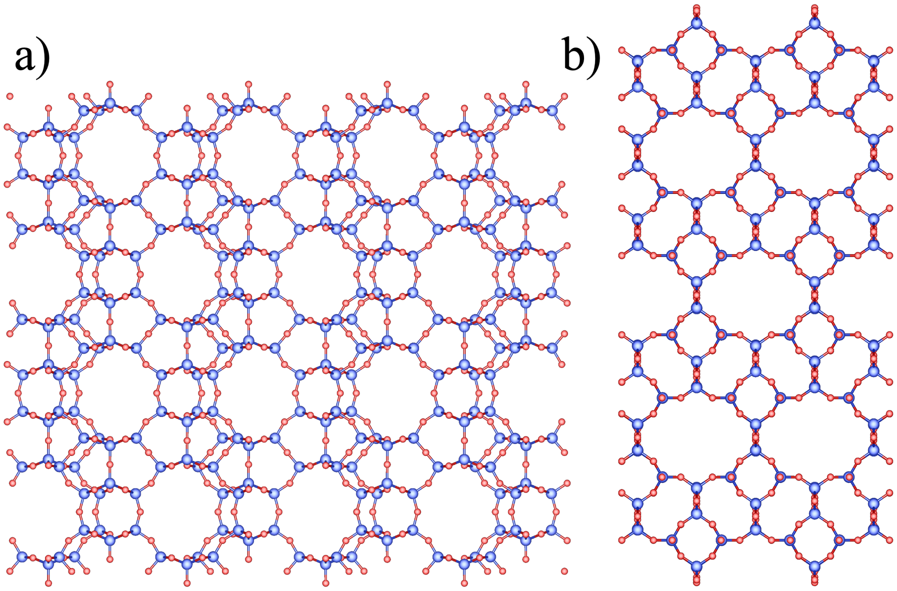

Figure 3: Structure of a supercell of the topology TER viewed along the [1 0 0] and [0 0 1] axes respectively, rotated for ease of inspection. Silicon is shown in blue, and oxygen in red.

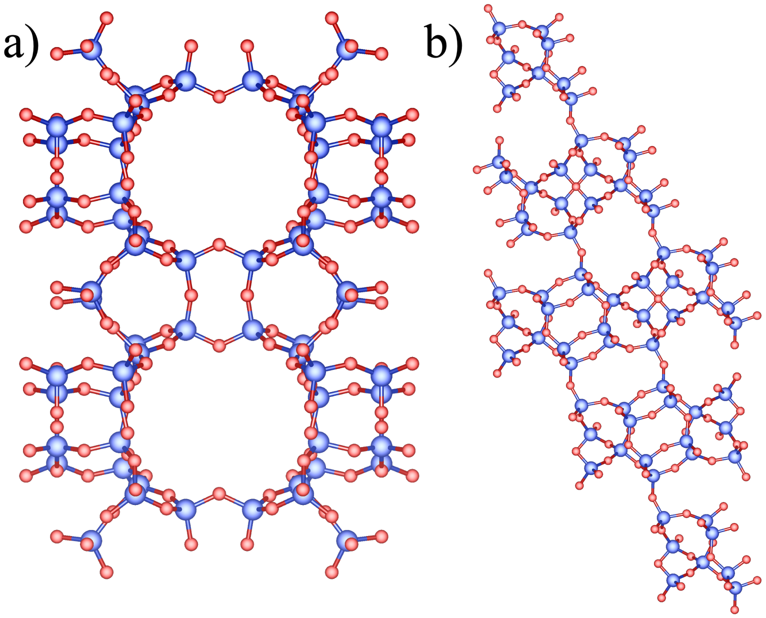

Figure 4: Structure of a supercell of the topology PCOD-8063931 viewed along the [1 0 0] and [0 1 0] axes respectively, rotated for ease of inspection. Silicon is shown in blue, and oxygen in red.

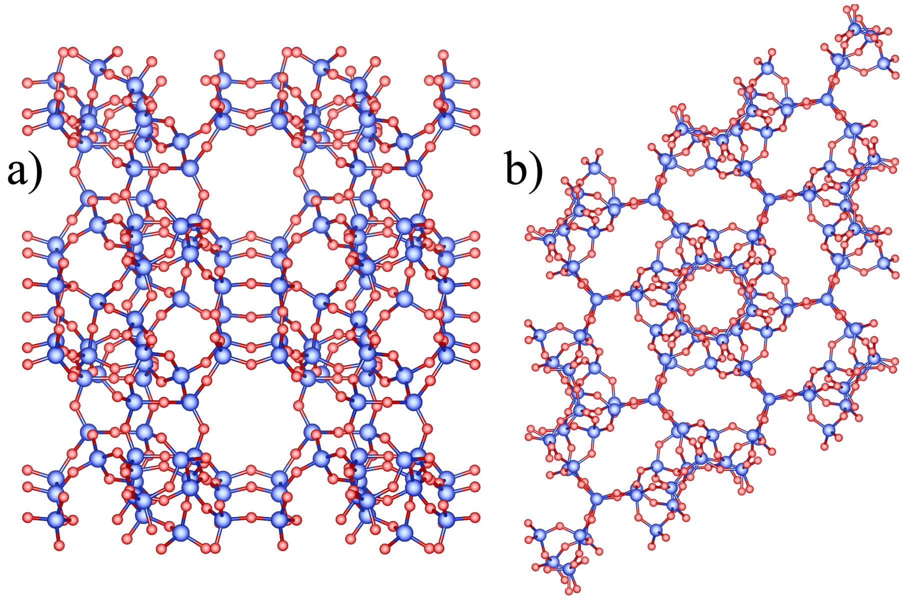

Figure 5: Structure of a supercell of the topology PCOD-8321668 viewed along the [0 1 0] and [0 0 1] axes respectively, rotated for ease of inspection. Silicon is shown in blue, and oxygen in red.

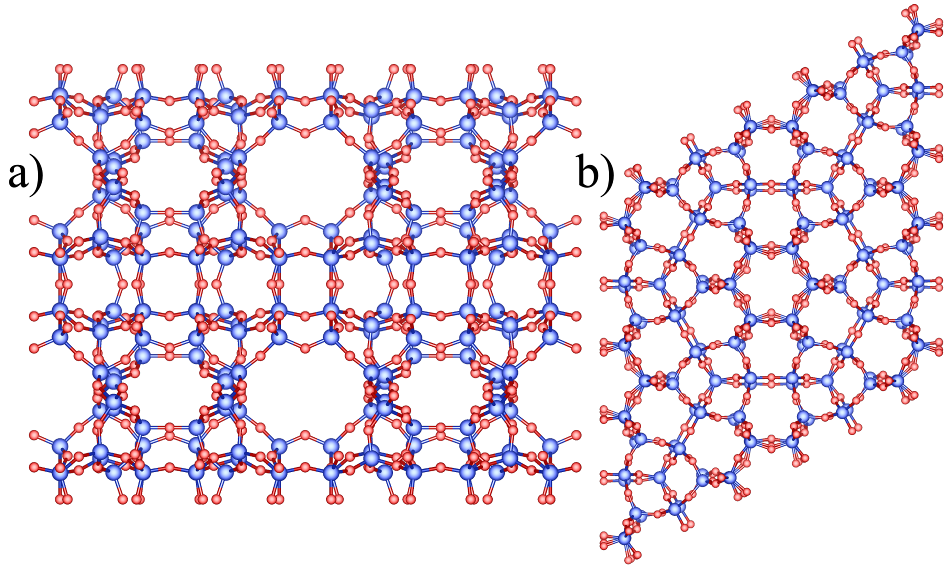

Figure 6: Structure of a supercell of the topology PCOD-8330068 viewed along the [0 1 0] and [0 0 1] axes respectively, rotated for ease of inspection. Silicon is shown in blue, and oxygen in red.

With respect to the three highest performing hypothetical structures shown in Figures 4, 5, and 6, the key structural feature responsible for the heightened performance of these frameworks is the shape of the channel network. In all three, sinusoidal channels are arranged in a hexagonal shape, allowing for the xylene ring to tightly fit at the intersections of the channel, with the methyl groups

pointing into the channels themselves. This finding is in good agreement with the results of Caro-Ortiz *et al.*[1] in their study of nine zeolite topologies. These adsorption patterns are shown clearly in the adsorption profiles available through this link.[2]

With reference to the known zeolite topologies studied, this hexagonal shaped channel structure is not present, which could be a deciding factor in the reduced selectivity for these frameworks. In MFI and TER, the multi dimensional channel network of sinusoidal channels allows for efficient packing of meta-xylene. Section 1 details how this particular channel network shape restricts the transport of meta-xylene in comparison to the other two isomers, and shows how MEL, a similar topology to MFI but with straight channels, shows a lower selectivity due to more favourable adsorption of para-xylene.

TON has a one dimensional channel system, and its adsorption behaviour is likewise dominated by its ability to preferentially accommodate meta-xylene in its sinusoidal channels. The shape of the channels allows optimal packing of meta-xylene, with an enthalpic penalty to the adsorption of para- and ortho-xylene in comparison. As seen in the main text of the article, some of the highest performing structures have one dimensional channel systems, whose improved selectivity over TON arises from a higher penalty to the adsorption of para-xylene, with more pronounced zig-zag patterns in the channel system.

1.1 Comparison of MFI and MEL

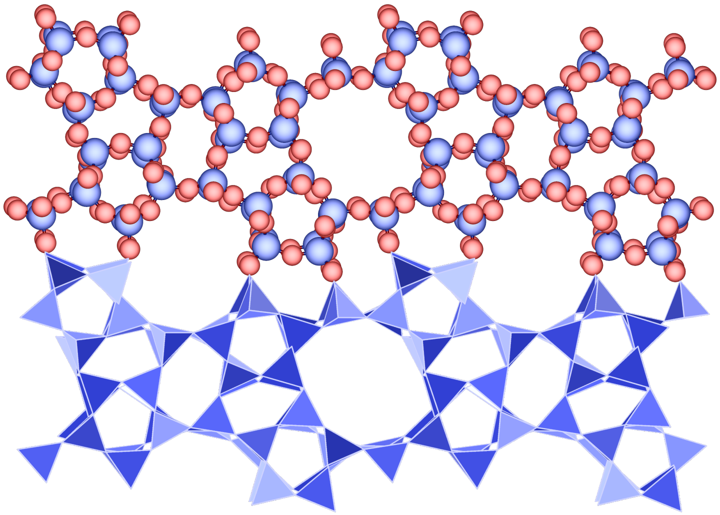

Figure 7: Structure of the siliceous form of the zeolite MFI shown as top) ball and stick form, atoms shown with their van der Waals radii and bottom) polyhedral form to show the silica tetrahedra. The structure is viewed along the [010] axis and shows a fragment cut from the extended solid. Silicon atoms and tetrahedra are shown in blue, and oxygen atoms are shown in red.

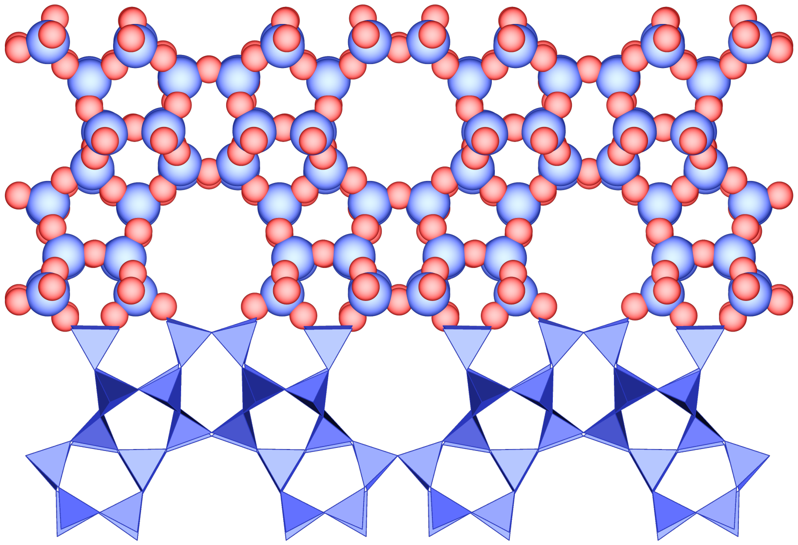

Figure 8: Structure of the siliceous form of the zeolite MEL shown as top) ball and stick form, atoms shown with their van der Waals radii and bottom) polyhedral form to show the silica tetrahedra. The structure is viewed along the [010] axis and shows a fragment cut from the extended solid. Silicon atoms and tetrahedra are shown in blue, and oxygen atoms are shown in red.

The two topologies MEL and MFI (see Figures 7 and 8 respectively) are both notable for having very similar structures, both being built from twelve unique tetrahedral (T) atoms (here, silicon). These twelve T atoms form pentasil layers; in MFI these layers are related by inversion, whereas in MEL they are related by mirroring. In MFI this gives rise to zig-zag channels parallel to the a axis, and straight channels parallel to the b axis, while in MEL this arrangement yields straight channels along the a and b axes. Due to the similarity between the two structures, it is worth asking the question as to why MFI showed such high selectivity in the meta-xylene isomerisation reaction, while MEL did not.

Structure	Adsorbate	Loading / mol kg $^{-1}$
MFI	Ortho-xylene	0.006
	Meta-xylene	0.493
	Para-xylene	0.029
MEL	Ortho-xylene	0.071
	Meta-xylene	0.411
	Para-xylene	0.164

Table 1: Comparison of the adsorption behaviour of the three xylene isomers in the zeolites MFI and MEL.

The results from the CFC-MC calculations allowed for us to determine that both MFI and MEL showed similar loading values for both meta- and ortho-xylene, with the most remarkable difference in their adsorption behaviour coming from para-xylene. From Table 1 we can see that MEL was able to accommodate 0.164 mol kg^{-1} of para-xylene compared to the 0.029 mol kg^{-1} of MFI.

With reference to their structures, this difference can be rationalised by the channel shapes of the two materials. Similarly to the high-performing hypothetical materials we have examined, MFI's zig-zag channel structure is able to allow the aromatic group of meta-xylene to adsorb strongly in the zig-zag nodes, with the methyl groups pointing into the edges. On the other hand, MEL has straight channels, and so is able to accommodate meta-xylene at channel intersections, but is also able to accommodate para-xylene favourably in the channels themselves. This difference in channel shape can thus be shown to be responsible for the difference in the materials' performance, and shows how these structure-property relationships can be powerful tools in guiding synthesis for optimal catalysts.

1.2 Loading and selectivity data

2 Computational Details

2.1 GCMC

All RASPA files are available through this link, showing examples of molecule files, force field files, pseudo atoms, and a simulation input file for the CFC-MC simulations.

2.2 Molecular Dynamics

Table 4 and 5 shows the tabulated results for the molecular dynamics simulations at 1223 K for the 2 and 3D hypothetical structures, as well as MFI. The order N MSD is shown in its raw form from the simulations whereas in the image in the main text it is log10 transformed. These simulations were run 10 times for 100 nanoseconds each, and the mean of the MSD for these 10 runs is shown.

The structure which showed the greatest diffusion of para-xylene underwent further molecular dynamics simulatons in order to determine a temperature at which its performance was equal to MFI's performance at 1223 K. This is in order to give an estimate as to the percentage reduction in temperature that this structure would allow, while maintaining the same selectivity and activity as MFI. These simulations were run for 100 ns as well. Since the MSD of meta- and ortho-xylene was trivially low for these structures, we only sought to find structures with an MSD of para-xylene greater than that of MFI. 10 simulations per structure were run at temperatures from 1223 K downwards in steps of 100 K in order to find the lowest temperature at which these structures outperformed MFI.

	Loading / mol kg^{-1}			
Framework Name	Ortho-xylene	Meta-xylene	Para-xylene	Selectivity
8153395	0.000014	0.208888	0.000059	2474.608439
8304211	0.000116	0.287876	0.000013	1930.208854
8192542	0.00018	0.206703	0.000111	613.88164
8234953	0.000252	0.35198	0.000488	411.645143
8294334	0.000486	0.247417	0.000085	375.628269
8326107	0.000066	0.390116	0.000849	369.109618
8182279	0.000103	0.205062	0.000431	332.268152
8234449	0.000448	0.411077	0.000725	303.383217
8300828	0.001052	0.457654	0.000372	278.08689
8324947	0.000602	0.319269	0.000552	239.558114
8295817	0.000716	0.47607	0.001259	208.690074
8162380	0.000782	0.331849	0.000716	191.753047
8162607	0.0001	0.232953	0.001271	147.117953
8118253	0.000797	0.307366	0.001282	127.967274
8122465	0.000657	0.290369	0.002341	83.856342
8134003	0.002213	0.404361	0.002138	80.458176
8120915	0.00069	0.304184	0.003002	71.326517
8282411	0.000333	0.488563	0.005638	70.837163
8302163	0.000595	0.353084	0.004132	64.654673
8078462	0.000109	0.219843	0.002913	62.981747
8053163	0.000118	0.216388	0.002898	62.110414
8082742	0.001642	0.390675	0.004147	58.420401
8145624	0.000126	0.307714	0.00447	57.968923
8133140	0.002131	0.315188	0.002671	56.830297
8322162	0.000243	0.325912	0.004928	54.554846
8140263	0.000185	0.443176	0.007202	51.933992
8282410	0.000621	0.520492	0.009114	46.283125

Table 2: Loading and Selectivity data for the top 81 structures.

The results are tabulated in Table 6.

Further reductions in temperatures resulted in a decrease in diffusion of para-xylene to below the level of MFI.

	Loading / mol kg^{-1}			
Framework Name	Ortho-xylene	Meta-xylene	Para-xylene	Selectivity
8174633	0.002748	0.651516	0.010225	43.475637
8306225	0.00315	0.230511	0.001568	42.291522
8067175	0.000157	0.272368	0.005425	42.235367
8215189	0.0054	0.435843	0.00467	37.467809
8151967	0.001862	0.344797	0.006242	36.83276
8321505	0.001449	0.444593	0.00974	34.397402
8083598	0.002056	0.264578	0.004782	33.491981
8049254	0.002163	0.201458	0.003086	33.219719
8300211	0.003216	0.218026	0.002803	31.358043
8133318	0.001479	0.259677	0.005707	31.281122
8193408	0.005251	0.727923	0.015336	30.609195
8152865	0.001617	0.353268	0.008417	30.478364
8306267	0.002093	0.250628	0.006121	26.414858
8268546	0.005468	0.444322	0.009228	26.173654
8319248	0.005046	0.802721	0.021551	26.126475
8215101	0.009345	0.386526	0.003469	26.112973
8321561	0.00131	0.49329	0.015299	25.709922
8156815	0.000012	0.253043	0.008651	25.285187
8095924	0.000526	0.260718	0.008495	25.020291
8248315	0.005287	0.527337	0.013171	24.731885
8133023	0.010438	0.557299	0.009821	23.813724
8330068	0.000015	0.206901	0.008258	21.649057
8227868	0.008304	0.764767	0.023006	21.144626
8094730	0.005867	0.466558	0.013384	20.979863
8295569	0.004903	0.49243	0.015553	20.839083
8251803	0.011747	0.296385	0.000602	20.775793
8301864	0.007939	0.424809	0.010355	20.101336
8325754	0.002208	0.257008	0.008894	20.039875
8281876	0.011068	0.593275	0.01473	19.907405
8167472	0.000344	0.440505	0.0194	19.314352

Table 3: Loading and Selectivity data for the top 81 structures continued.

	Loading / mol kg^{-1}			
Framework Name	Ortho-xylene	Meta-xylene	Para-xylene	Selectivity
8110796	0.000185	0.215053	0.009587	19.051691
8224932	0.006748	0.403402	0.012224	18.407627
8223024	0.002411	0.242428	0.009395	17.775991
8063931	0.002522	0.298706	0.012031	17.768086
8282464	0.00105	0.228024	0.010185	17.569071
8215290	0.007571	0.376143	0.011216	17.331906
8165995	0.024791	1.122487	0.031313	17.319842
8133881	0.01558	0.449831	0.007174	17.113904
8305940	0.0001	0.400199	0.020355	16.936339
8143363	0.003123	0.353286	0.01506	16.820071
8261866	0.004466	0.503576	0.022418	16.214959
8181417	0.011329	0.388987	0.009479	16.183015
8222628	0.000012	0.23845	0.012905	15.980824
8321668	0.018459	0.655294	0.017132	15.938586
8141101	0.001919	0.371289	0.018276	15.915461
8232569	0.000645	0.338938	0.01842	15.389991
8220059	0.002598	0.32717	0.015864	15.340623
8165792	0.015447	0.37037	0.005937	14.993763
8263582	0.000956	0.319799	0.017526	14.979026
8253718	0.023967	0.440155	0.001566	14.923222
8234885	0.009623	0.488433	0.018749	14.903085
8217007	0.020038	0.420665	0.004468	14.860196
8316000	0.00003	0.285965	0.018269	13.527978
8230305	0.010306	0.255288	0.00741	12.474505

Table 4: Loading and Selectivity data for the top 81 structures continued.

3 Neural Network details

3.1 Model architecture

Both the classification and regression models were trained using the TensorFlow implementation of the Keras library.[3] Prior to training, 10% of the data was reserved as a test set, and 20% as validation data in order to implement early stopping to prevent overfitting to the training data. For the classification model, in both of these data splits stratified splitting was used in order to ensure

Structure	Adsorbate	MSD / Å^2
MFI	Ortho	1.7
	Meta	2.1
	Para	2263.9
8263582	Ortho	3.5
	Meta	130.1
	Para	34243.8
8063931	Ortho	106.3
	Meta	32.3
	Para	201976.4
8281876	Ortho	1649.9
	Meta	18.6
	Para	42468.0
8162380	Ortho	1.0
	Meta	1.4
	Para	395.5
8330068	Ortho	4.5
	Meta	0.4
	Para	52666.3
8268546	Ortho	0.4
	Meta	3.7
	Para	2691.9
8261866	Ortho	0.9
	Meta	2.5
	Para	53.8
8321668	Ortho	34.5
	Meta	12.1
	Para	53143.6
8324947	Ortho	0.5
	Meta	0.4
	Para	2.3

Table 5: MSD data for MFI and the 2D hypothetical structure's MD simulations at 1223 K, run for 100 ns.

Structure	Adsorbate	MSD / Å^2
MFI	Ortho	1.7
	Meta	2.1
	Para	2263.9
8165995	Ortho	7.4
	Meta	425.6
	Para	21188.8
8304211	Ortho	0.6
	Meta	1.3
	Para	13126.1
8316000	Ortho	0.7
	Meta	0.7
	Para	3.1

Table 6: MSD data for MFI and the 3D hypothetical structure's MD simulations at 1223 K, run for 100 ns.

Temperature / K	MSD / Å^2
923	3393.1
823	2760.1
723	2112.0
623	1647.6

Table 7: MSD data for MD simulations of structure 8063931 in steps of 100 K for the self-diffusion of para-xylene.

that a representative number of highly selective structures were present in the training set. The data for both models was normalised prior to training using a Keras normalisation layer.

3.2 Classification model

The model contained 3 fully connected hidden layers with 949 neurons, each with batch normalisation, 'relu' activation, and dropout with a rate of 50%. This network was trained with binary cross-entropy as the cost function, with early stopping implemented when this metric stopped decreasing for the validation set. The Adam optimiser, which has been shown to be robust for a variety of cases, was used, along with a biased initialisation in order to reduce training times.[4]

In order to prevent overfitting of the model to the over-represented class, class weights were used

which allow the neural network to, in effect, pay more attention to the under represented class. Similarly, as batch training was used, the batch size was chosen to be sufficiently large so that each batch would likely contain structures from the under represented highly selective class.

3.3 Regression model

The regression model had a similar architecture to the classification model, but with 2 fully connected hidden layers each with 433 neurons. Similarly, each dense layer used batch normalisation, a 'relu' activation function, and dropout with a rate of 50%. The Huber loss was chosen as the loss function with the Nadam optimiser which is similar to the Adam optmiser used for classification but with Nesterov momentum.[5]

3.4 Descriptors

Topological descriptors were calculated using persistent homology. A similar workflow to the excellent work of Krishnapriyan *et al.*[6] was used, using Diode to generate the alpha shapes, then Dionysus 2 in order to calculate persistence diagrams. Finally PersIm was used to convert these into persistence images. We scaled our persistence images to a 50x50 resolution, scaled by the maximum birth and persistence values, as done in their work. We employed a sigma value (the spread of the Gaussian's transforming the persistence diagrams into persistence images) of $\sigma = 0.2$. For a more in-depth explanation of these descriptors we refer the reader to their work.[6]

PoreBlazer was used to calculate textural descriptors. An example of an output file from a PoreBlazer calculation for MFI is shown below, indicating the textural descriptors we used in our study for the training of neural networks.

Zeo++ was used to calculate the pore limiting diameter and largest cavity diameter for the initial screening of structures based on their PLD being greater than 4 Å. These values were also used as descriptors for training the neural networks.

References

[1] Ortiz, S. C., Zuidema, E., Rigutto, M., Dubbeldam, D. & Vlugt, T. J. Competitive adsorption of xylenes at chemical equilibrium in zeolites. *Journal of Physical Chemistry C* **125**, 4155–4174 (2021). URL `https://pubs.acs.org/doi/abs/10.1021/acs.jpcc.0c09411`.

[2] Hewitt, D. Adsorption Profiles. URL `https://github.com/d4n-hewitt/d4n-hewitt`.

[3] Abadi, M. *et al.* TensorFlow: Large-scale machine learning on heterogeneous systems (2015). URL `https://www.tensorflow.org/`. Software available from tensorflow.org.

Descriptor	Value	Explanation
V_A^3	41690.253	Volume of the system, V, Å^3
M_g/mol	46145.280	Molecular weight of the unit cell, g/mol
RHO_g/cm^3	1.838	Density, g/cm3
PLD_A	4.00	Pore limiting diameter, Å
LCD_A	5.66	Largest cavity diameter, Å
D	1	Number of directions the system is percolated in
$S\_AC_A^2$	10759.84	Accessible surface area, $S(AC, T), \AA2$
$S\_AC_m^2/cm^3$	2580.90	Accessible surface area, $S(AC, T), m2/cm3$
$S\_AC_m^2/g$	1404.20	Accessible surface area, $S(AC, T), m2/g$
$V\_He_A^3$	11042.451	Helium pore volume, $V(He, T), \AA3$
$V\_He_cm^3/g$	0.144	Helium pore volume, $V(He, T), cm3/g$
$V\_G_A^3$	17695.910	Geometric pore volume, $V(G, T), \AA3$
$V\_Gcm^3/g :$	0.231	Geometric pore volume, $V(G, T), cm3/g$
$V\_POA^3 :$	16993.383	Probe-occupiable pore volume, $V(PO, T), \AA3$
$V\_POcm^3/g :$	0.222	Probe-occupiable volume, $V(PO, T), cm3/g$
FV_PO:	0.40761	Volume fraction, , $V(PO, T)/V$
$S\_AC\_A^2$	10455.33	Network-accessible surface area, $S(AC, A), \AA2$
$S\_AC\_m^2/cm^3$	2507.86	Network-accessible surface area, $S(AC, A), m2/cm3$
$S\_AC\_m^2/g$	1364.46	Network-accessible surface area, $S(AC, A), m2/g$

Table 8: PoreBlazer output file with explanations

[4] Kingma, D. P. & Ba, J. L. Adam: A method for stochastic optimization. In *3rd International Conference on Learning Representations, ICLR 2015 - Conference Track Proceedings* (International Conference on Learning Representations, ICLR, 2015). URL `https://arxiv.org/abs/1412.6980v9`. `1412.6980`.

[5] Dozat, T. Incorporating Nesterov Momentum into Adam. *ICLR Workshop* 2013–2016 (2016). URL `http://mattmahoney.net/dc/text8.zip`.

[6] Krishnapriyan, A. S., Haranczyk, M. & Morozov, D. Topological Descriptors Help Predict Guest Adsorption in Nanoporous Materials. *The Journal of Physical Chemistry C* **124**, 9360–9368 (2020). URL `https://pubs.acs.org/doi/full/10.1021/acs.jpcc.0c01167`. `2001.05972`.
